# miR-155-3p: processing by-product or rising star in immunity and cancer?

**DOI:** 10.1098/rsob.220070

**Published:** 2022-05-25

**Authors:** Owen Dawson, Anna Maria Piccinini

**Affiliations:** School of Pharmacy, University of Nottingham, Nottingham NG7 2RD, UK

**Keywords:** miRNA strand selection, miRNA arm switching, isomiRs, miR-155-5p, miR-155-3p, immunity and cancer

## Abstract

MicroRNAs (miRNAs) are key players in gene regulation that target specific mRNAs for degradation or translational repression. Each miRNA is synthesized as a miRNA duplex comprising two strands (5p and 3p). However, only one of the two strands becomes active and is selectively incorporated into the RNA-induced silencing complex in a process known as miRNA strand selection. Recently, significant progress has been made in understanding the factors and processes involved in strand selection. Here, we explore the selection and functionality of the miRNA star strand (either 5p or 3p), which is generally present in the cell at low levels compared to its partner strand and, historically, has been thought to possess no biological activity. We also highlight the concepts of miRNA arm switching and miRNA isomerism. Finally, we offer insights into the impact of aberrant strand selection on immunity and cancer. Leading us through this journey is miR-155, a well-established regulator of immunity and cancer, and the increasing evidence that its 3p strand plays a role in these arenas. Interestingly, the miR-155-5p/-3p ratio appears to vary dependent on the timing of the immune response, and the 3p strand seems to play a regulatory role upon its partner 5p strand.

## Graphical abstract

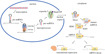

## 1. Introduction

Nearly three decades have passed since the discovery in 1993 of the small non-coding RNA molecule lin-4, the first in an ever-expanding group of post-transcriptional regulatory elements which have since been categorized as microRNAs (miRNAs) [1]. In the years since, many discoveries have been made that define the biology of miRNAs, with the development of powerful technologies such as RNA-Seq and complex molecular and biochemical approaches allowing examination of molecular, evolutionary and functional mechanisms previously difficult to determine. An example of such an advancement lies in our understanding and functional appreciation of the ‘star strand’ of the mature miRNA duplex and how regulation and selection of this strand occurs. Referred to as the miRNA*, this strand of the miRNA duplex generally features low cellular abundance, and in the past has been considered to have no biological activity of its own [2]. However, mounting evidence has revealed early aspersions on the functionality of the miRNA* strand to be incorrect, leading to closer examination of both miRNA strands in this new dual-functional context and a shift in nomenclature favouring the usage of 3p and 5p to refer to the strand arms. Herein, we shall outline the key research to date which provoked this shift in the non-functional miRNA* strand paradigm, as well as the related principles of miRNA arm switching and miRNA isomerism. Furthermore, we shall use this information in a balanced analysis of miRNA-155, a microRNA of substantial historical importance, being one of the first discovered oncomiRs as well as the first miRNA with a murine knockout mouse model [3–9]. miR-155 is a key miRNA in both immunity and cancer and its miRNA* strand, miR-155-3p, has been functionally implicated in both areas but is critically understudied, at least partly due to its lower cellular abundance compared to its partner strand.

## 2. MicroRNA biogenesis: overview

MiRNAs are approximately 22 nt long single-stranded RNA molecules, which direct the targeting of mRNA transcripts by the RNA-induced silencing complex (RISC), resulting in translational repression or decay of mRNA transcripts whose sequences are complementary to that of the miRNA. In the animal kingdom, binding of the miRNA to its target mRNA does not require perfect base pairing, allowing for a single miRNA to target multiple sequentially dissimilar mRNAs. However, base pairing of nucleotides 2–7 of the miRNA, a region known as the ‘seed sequence’, with the target mRNA (typically its 3′ UTR) is necessary [10]. This functionality has led to miRNAs being central components of core cellular processes such as development, differentiation, proliferation, inflammation and the stress response, while their deregulation can also influence the pathogenesis of multiple disorders such as arthritis, cardiac hypertrophy and Alzheimer's disease [11–15]. Due to this diverse functionality, mammalian miRNA biogenesis is a tightly coordinated, multi-step process, the many facets of which have been extensively and skilfully reviewed elsewhere [16].

**Figure 1.**
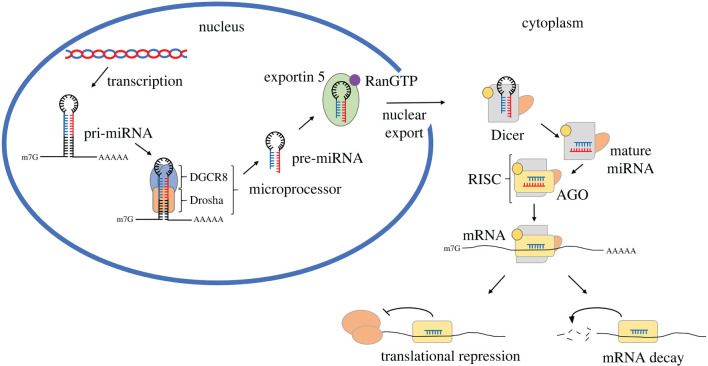
Classical miRNA biogenesis pathway. The primary miRNA transcript (pri-miRNA) is processed by the Microprocessor, consisting of Drosha and DiGeorge Syndrome Critical Region 8 (DGCR8) proteins. This produces a precursor miRNA (pre-miRNA) which is exported from the nucleus via Exportin 5 in a RanGTP-dependent mechanism. Within the cytoplasm, the pre-miRNA is processed by Dicer and its associated proteins to produce a mature miRNA duplex, which is loaded onto the RNA-induced silencing complex (RISC). Here, strand selection occurs with the retained strand targeting the RISC to complementary mRNA transcripts where it may perform its effector functions.

In brief, transcription of miRNA genes by RNA polymerase II produces a primary miRNA transcript (pri-miRNA), a single-stranded RNA molecule interspersed with one or more double-stranded hairpin regions within which the miRNA sequence resides [17]. In the cell nucleus, the hairpin region of the pri-miRNA is recognized and cleaved by the Microprocessor, a protein complex primarily consisting of the RNAse III enzyme Drosha and the ds-RNA binding protein DiGeorge Syndrome Critical Region 8 (DGCR8) [18–20]. The resultant double stranded RNA hairpin molecule is termed precursor-miRNA (pre-miRNA) and features 5′ phosphate and 3′ hydroxyl groups, and a 2 nt 3′ overhang (figure 1). Multiple other factors such as the DEAD-box RNA helicases DDX17 and DDX5 are thought to associate with the Microprocessor and regulate processing efficiency [21,22].

Once transported into the cytoplasm, the pre-miRNA is cleaved by the protein Dicer, which removes the pre-miRNA terminal loop, facilitating loading of the now mature miRNA onto the RISC. This cleavage process involves first the PAZ domain of Dicer that interacts preferentially with the pre-miRNAs 3′ overhang. Cleavage then occurs 21–25 nt upstream of the bound end, using an RNase III catalytic domain to introduce a staggered break and remove the terminal loop (figure 1) [23–26]. Importantly, the site of this cleavage has been directly linked to an event known as arm-switching, as a thermodynamically unstable 5′ pre-miRNA end can instead be bound by the PAZ domain of Dicer, leading to a different cleavage site due to the staggered nature of the pre-miRNA termini [27]. It has been shown that modifications to the terminal pre-miRNA ends that influence PAZ binding can thus determine the production of iso-miRs and alternate strand synthesis.

## 3. The miRNA duplex and strand selection

Throughout miRNA biogenesis, the mature miRNA sequence exists as a duplex structure, arising from the initial folding of the pri-miRNA transcript into stem loops. This protects the sequence from degradation and facilitates interactions with miRNA processing enzymes such as Drosha and Dicer, both of which feature dsRNA binding domains. However, as only one miRNA strand is used as a guide for the RISC, a process of strand selection is necessary to determine which strand RNA sequence is used and which is discarded. This process of strand selection has been expertly outlined elsewhere [28].

Evidence suggests that this process occurs during the loading of the mature miRNA onto the Argonaute (Ago) protein, an essential component of the RISC. Specifically, the 5′ end of the retained miRNA strand interacts with a binding pocket in the Ago protein that is located at the interface between its MID (middle) and PIWI domains, while the 3′ end fits into a hydrophobic cavity within the PAZ domain [29–36]. The strand that binds to this pocket, either the 5′ or 3′ strand, denoted as 5p and 3p, respectively, is selected via two criteria. The first selection criterion is based on the thermodynamic features of each miRNA duplex end, with Ago showing a tendency to incorporate the strand with the lowest 5′ end internal stability, probably due to increased access given to the MID/PAZ binding pocket, thought to be facilitated by regions such as the PAZ phosphate binding pocket [37–39]. The second criterion involves the identity of the 5′ terminal nucleotide of the miRNA strands, selected via a nucleotide specificity loop found within the MID domain of Ago [39]. In the case of human Ago2, this bias is expressed via a preference for 5′ terminal uridine monophosphate (UMP) and adenosine monophosphate (AMP), with an affinity approximately 20 times greater than that for cytidine monophosphate (CMP) and guanosine monophosphate (GMP), which both sterically clash with the specificity loop in the MID domain [31,33]. Together, these two criteria dictate a strand selection process that results in the asymmetrical functional utilization of the miRNA 5′ and 3′ strands. However, these criteria do not account for all miRNA strand asymmetry, with the removal of key amino acids within *C. elegans* Ago-like protein not inhibiting all strand selection [40]. This was furthered by recent bioinformatic analysis of both miRNA strands, identifying that 17–25% of miRNAs examined did not follow either of these selection criteria [28].

## 4. miRNA arm relevance and miRNA*

It was noted early in the study of miRNAs that asymmetrical strand abundance was common, with sequencing efforts identifying an accumulation of one miRNA strand but frequently being unable to identify its antisense strand, presumed to be due to its degradation [2]. An example of this is let-7, with the failure to detect its antisense strand through *in vitro* assays, leading the investigators to conclude its miRNA* to be not generated or to have low stability [23]. This led to the denotation of the strand within the miRNA duplex that accumulates to a higher steady-state level as the mature miRNA (e.g. miR-155), while the less abundant antisense strand was labelled the miRNA* strand (e.g. miR-155*). Although this proved a sensible approach for strand categorization in a time when the number of identified miRNAs increased 10-fold from 2004 to 2008, attached to this nomenclature were false assumptions as to the functionality of the miRNA* strand, with the belief that miRNA* strands were solely a structural requirement for the processing of the miRNA [41,42].

The assumption that the miRNA* is not functional was majorly challenged in 2008 when Okamura *et al.* identified that approximately 1/5 of the 132 miRNAs* examined in *Drosophila* had conserved 3′ UTR mRNA targets across related species [43]. Additionally, association of the miRNA* with Ago was demonstrated, illustrating a functional ability that was further validated via miRNA* strand-mediated repression of target site constructs *in vitro* [43]*.* In 2011, Yang *et al.* presented similar evidence for miRNA* strand functionality in vertebrates, expanding upon previous work by identifying a 5′ terminal bias against G in both miRNA and miRNA* strands, implying that both strands are under a similar selective pressure for properties favourable to strand selection [44]. This study also made apparent the degree of miRNA* strand seed sequence conservation, being less than that of the predominant strand, but greater than the surrounding nucleotides. Notably, by-products of small noncoding RNAs have been proposed as functional in other classes of noncoding RNAs, illustrating a conserved avoidance of transcriptional and functional waste in complex organisms. For example, snoRNA-derived small RNAs (sdRNAs) are fragments of small nucleolar RNAs (snoRNAs) that have shown emerging functions in splicing regulation in disorders such as Prader Willi syndrome [45,46]. Together, these studies have shifted the perception of miRNA*, resulting in a change in nomenclature away from the functionally definitive designations of miRNA and miRNA* and towards the more descriptive miRNA-5p and miRNA-3p.

The functional relevance of the less abundant miRNA* strand has since been made apparent for a number of miRNAs. For instance, the miR-574* strand (miR-574-5p) has been shown to be overexpressed in advanced gastric cancer, leading to cell proliferation, the opposite function of its partner strand (miR-574-3p), while miR-21* (miR-21-3p) has been shown to have tumour suppressive qualities in ovarian cancer [47,48]. The participation of miRNA* in regulatory networks furthers miRNA functional diversification as the miRNA* seed sequence can target different mRNAs than those targeted by its partner strand, adding an increased array of potential mRNA targets. However, it should be noted that there is some targeting overlap between miRNA* and non-miRNA* of different mature miRNAs as well as some cases of both miRNA strands sharing an mRNA target [44,47].

It is important to note that within the designation of miRNA* lies a large variability in strand abundance, with different miRNA:miRNA* ratios. Although some miRNA* strands are lower in abundance than their partner strands, they may still have a higher abundance than the predominant strand of other functionally categorized miRNAs. For example, although let7b* (let-7b-3p) has a considerably lower expression than its partner strand, its documented reads of approximately 19 672 on miRBase place it well above the functional threshold of 1000 copies per cell that is used as a universal cut-off for miRNA functional activity by web servers such as TargetScan. Conversely, some miRNA* strands have such a low abundance that they arguably do not have a biologically relevant function. Examples of these are miR-155* (miR-155-3p) and miR-100* (miR-100-3p), both of which are reported in miRBase with a deep sequencing depth less than 300 reads. Such links between miRNA abundance and functionality are key to our understanding of potential miRNA* activity. Studies of mammalian Ago2-RISC kinetics indicate that low abundance miRNAs would have limited biological impact due to their reduced stochastic incidence of mRNA targeting as well as competition for Ago2 incorporation by higher abundance miRNAs, meaning they could only have a small impact on their target mRNA [49]. However, this is further complicated by two factors. Firstly, miRNAs show differing degrees of subcellular localization, leading to the whole cell miRNA abundance not necessarily reflecting local miRNA ratios. For example, cellular compartments such as the nucleus, endoplasmic reticulum and mitochondria have been shown to have distinct miRNA populations, as well as membrane-less compartments, including stress granules and processing bodies [50]. Secondly, the miRNA* strand designation is often assigned based upon steady-state miRNA levels, both these factors being in part artefacts of early miRNA studies wherein whole organism RNA extracts were utilized in experiments [1]. This is in conflict with the present understanding of miRNA regulation as being highly dynamic, with strand ratios and quantities changing dependent on various factors governing a phenomenon known as arm switching.

## 5. miRNA arm switching

Coupled with the expansion of our understanding of miRNA* strand functionality is the growing comprehension and appreciation of miRNA arm switching. This is a phenomenon whereby the strand ratio of miRNA-5p and -3p from the same mature miRNA can change between cell and tissue type, developmental stage and pathological state. Arm-switching and its regulation has recently been thoroughly reviewed [28]. Such regulation often involves the synthesis of isomiRs, mature miRNA strands whose RNA sequences are different to that of their genomic sequence [51–54]. IsomiRs have been confirmed to functionally associate with the RISC and mRNA targets [55,56]. For instance, an isomiR of miR-376 aids uric acid homeostasis through regulatory targeting not shared by the canonical form and, similarly, an isomiR of miR-140-3p regulates the cholesterol pathway by targeting unique to this isomiR [57]. Generation of isomiRs has the potential to change the strand ratio as modifications to the 5′ sequence and structure of the mature miRNA leads to changes in stability of both ends, thus influencing strand selection by RISC. However, evidence suggests that miRNA arm switching events are not solely controlled by the mature strands thermodynamic properties, with expression of *Tribolium* and *Drosophila* miRNA-10 transcript within the same cell line finding each to lead to a different strand preference, even though both have an identical mature sequence [58].

Template isomiRs are produced by altered cleavage of the miRNA by Drosha or Dicer, while non-template isomiRs are the result of RNA remodelling factors acting upon the miRNA ends. The regulation of miRNA biogenesis co-factors is a likely mechanism by which template isomiRs may arise, with the Dicer associated factor TRBP and Drosha associated co-factors DGRC8 and DDX5/17 potentially influencing cleavage location [59–64]. The protein Adenosine Deaminase Acting on RNA (ADAR) has been implicated in non-template isomiR generation via knockout experiments in mice, with the protein causing adenine to inosine deamination which has been shown to influence strand selection [65–67]. More recently, the role of 3′ terminal uridyl transferase 4-7 (TUT4-7) in generating isomiRs has been established, with 3′ uridylation of miR-324 leading to a shift in the Dicer cleavage site that influences end architecture and leads to an arm switching event as miR-324-3p becomes the more abundant strand [27].

As well as arm switching, other miRNA regulatory processes could also be responsible for changes in miRNA strand ratio between tissue types and conditions. There is evidence for the increased availability of mRNA targets leading to stabilization of cognate miRNA strands in a process called target-mediated miRNA protection (TMMP) [68]. Thus, a ratio shift in favour of the miRNA* strands could be the result of an increase in expression of their mRNA targets. Inversely, target-directed miRNA degradation (TDMD) is also a recognized phenomenon whereby binding of the miRNA strand to a specific target mRNA leads to an increase in the decay rate as these targets promote exposure of the miRNA 3′ end to degradation inducing modifications [69–72]. Additionally, circular RNAs (circRNAs) have been shown to sponge miRNAs by presenting multiple binding sites along their lengths, with changes in their expression or TDMD targets also likely to influence strand ratio and arm switching events [73].

Recent studies such as that by Kim & Kim [27] highlight the stringently regulated nature of miRNA arm switching, providing a potential means by which many low abundance miRNA* strands could attain functional relevance in specific tissue types or pathological states [27]. miR-155-3p is an example of such a miRNA* strand, which, despite having a documented low expression compared to its partner strand, displays obvious functional relevance in specific tissue types, conditions and time frames.

## 6. MiR-155: molecular characteristics

### 6.1. Pri-miR-155 processing

**Figure 2.**
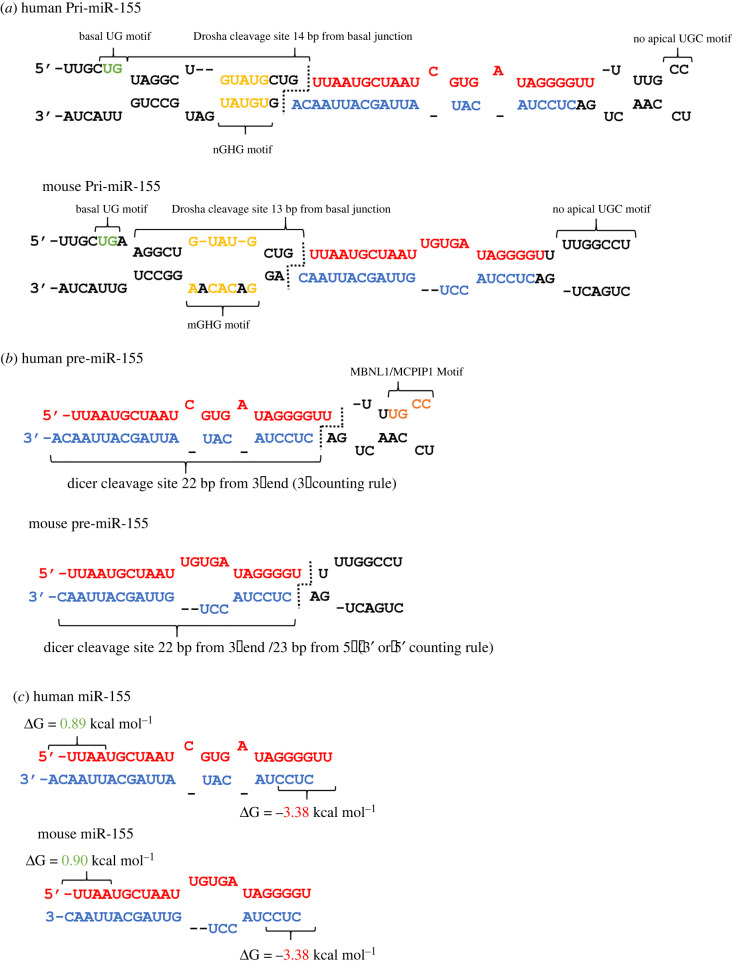
miR-155 biogenesis motifs and cleavage sites. (*a*) Predicted structure of human and murine pri-miR-155 via the RNAstructure web tool [76]. Basal UG motif (green), predicted mGHG motif (yellow) and lack of apical UGU motif are labelled alongside the Drosha cleavage site. (*b*) Predicted structure of human and murine pre-miR-155 via the RNAstructure web tool. Predicted pre-miRNA processing motifs are labelled. Cleavage site and predicted ‘counting rule’ utilized by Dicer is shown [77]. (*c*) The human and murine miR-155 duplex, featuring the miR-155-5p strand (red) and the miR-155-3p strand (blue). Nucleotides used for predictions of 5′ and 3′ end stability via RNAcofold are indicated [78,79].

Pri-miR-155 is transcribed from a monocistronic locus within the host B-cell Integration Cluster *(BIC)* located on chromosome 21 [74,75]. Secondary structure prediction and motif analysis can provide insight into the interactivity of the pri-miR-155 transcript with the Microprocessor (figure 2). Three motifs have been identified as key to pri-miRNA interactions with the core Microprocessor components Drosha and DGCR8. These being an apical UGU motif, which facilitates DGCR8 interaction, an mGHG motif, which guides Drosha cleavage site determination, and a basal UG, which orientates the pri-miRNA and influences Drosha-mediated cleavage [80–83]. Human pri-miR-155 does not feature an apical UGU motif, while its mGHG motifs have been predicted by Chul Kwon *et al*. to have no significant effect on cleavage site determination [83]. Thus, based on current knowledge, its basal UG motif is the only key site of cleavage determination present (figure 2*a*). Its location 2 nt upstream of the predicted basal junction leads to the possibility of alternate cleavage events, with the Drosha known to cleave both 13 bp upstream of the basal junction and 14 bp upstream of the UG motif, depending on the contribution of other motifs (figure 2*a*) [83]. In addition, the lack of a CNNC motif 17–18 nt downstream of the cleavage site makes regulation of pri-miR-155 processing by the Microprocessor co-factors DDX17 and SRF3 unlikely [80,84]. Overall, these factors, coupled with the predicted structural features of pri-miR-155 such as its small apical loop of 4 nt, present a pri-miRNA with a reduced accuracy and efficiency of processing as a result of sparse microprocessor interactivity (figure 2*a*) [84]. It could be speculated that this is advantageous, acting as a buffer against mis-regulated miR-155 transcription, the detrimental consequences of which being apparent in the known oncogenic and chronic inflammatory functions of miR-155-5p [85,86].

### 6.2. Pre-miR-155 processing

Pre-miR-155 cleavage by Dicer seems to occur via the 3′ counting rule, with Dicer being anchored to the 3′ end of the pre-miR-155 duplex and cleaving 22 nt upstream of this end [77]. This end selection is due to the lower relative stability of the AC overhang at the duplexes 3′ end, compared to the U-A bond at the 5′ end (figure 2*b*) [77]. Small inaccuracies in pri-miRNA cleavage have little potential to change the pre-miRNA cleavage site due to the 2 nt overhang at the 3′ end resulting in a consistently lower stability than the paired 5′ end. Pre-miRNA end modification has been documented to affect Dicer cleavage sites, introducing the possibility that miR-155 synthesis may also be modified in this manner [27]. Additionally, the apical loop of human pre-miR-155 features binding motifs for both MBNL1 and MCPIP1. Specifically, the former reduces miRNA synthesis by blocking Dicer binding, while the latter directly cleaves the pre-miRNA preventing further processing, an additional layer to miR-155 biogenesis regulation (figure 2*b*) [87,88]. Notably, mouse pre-miR-155 lacks both MBNL1 and MCPIP1 binding motifs.

### 6.3. miR-155 strand selection

When examining the human miR-155 mature duplex using the strand selection criteria, the reason behind the high relative expression of the 5p strand compared to the 3p strand becomes apparent. The 5′ terminal UMP of the 5p strand is known to have an Ago binding affinity approximately 20 times greater than that of the CMP nucleotide on the 5′ terminus of the 3p strand (figure 2*c*) [33]. Coupled with this is the lower relative thermodynamic stability of the 5′ end of the 5p strand (ΔG = 0.89 kcal mol^−1^; calculated by RNAcofold), compared to that of the 3p strand (ΔG = −3.38 kcal mol^−1^) (figure 2*c*) [78,79]. This demonstrates the general stability of miR-155 strand selection, with only significant structural or sequence changes beyond the miRNA ends likely influencing strand selection and causing miR-155-3p arm switching events.

### 6.4. miR-155 isomiRs and arm switching

A number of tools, which operate using different alignment strategies, to manage cross-mapping events, abundance cutoffs and/or isomiR annotation methods, have been developed to analyse miRNAs and their respective isomiRs. For instance, IsomiR Bank, a collection of greater than 300 000 isomiRs detected in more than 2700 RNA samples from eight different species subjected to small RNA next-generation sequencing analysis, reports the various isoforms of miRNAs that have been generated as a result of imprecise and alternative Drosha/Dicer cleavage or addition of non-templated nucleotides [89]. In the case of miR-155, the database reports 13 miR-155-3p isomiRs occurring in humans, the most abundant of which being expressed at level equal to or above that of the canonical miR-155-3p (table 1). These highly expressed isomiRs include cancerous tissues such as breast cancer and renal cell carcinoma, in which miR-155-3p has been shown to exert a regulatory function selection [90–94]. While the majority of these miR-155-3p isomiRs feature 5′ or 3′ strand deletions, which could affect strand selection, there is no abundance of isomiRs that would be typical of a documented arm switching mechanism such as 3′ uridylation [27] (table 1).

**Table 1.** Summary of miR-155-3p isomiRs. miR-155-3p isomiR sequences from IsomiR Bank, their highest expressing tissue and reads per million within that tissue [89]. Changes to the RNA sequence (red) and deletion events (*) are depicted.

miR-155-3p sequence	highest expressing tissue (IsomiR Bank)	reads per million
**canonical miR-155-3p**		
3′-ACAAUUACGAUUAUACAUCCUC	centroblast - tonsils	2.92
**miR-155-3p isomiR**		
3′-ACC**UACGAUUAUACAUCCUC	clear cell renal cell carcinoma	3.26
3′-*CAAUUACGAUUAUACAUCCU*	invasive ductal carcinoma - breast cancer	2.76
3′-ACAAUUACGAUUAUACAUCCU*		2.59
3′-***ACUACGAUUAUACAUCCUC	clear cell renal cell carcinoma	1.65
3′-**AAUUACGAUUAUACAUCCU*		1.45
3′-***AUUACGAUUAUACAUCCUC	invasive ductal carcinoma - breast cancer	1.37
3′-******ACGAUUAUACAUCCUCA	clear cell renal cell carcinoma	1.22
3′-******ACGAUUAUACAUCCUCAG		0.90
3′-*CAA*UACGAUUAUACAUCCUC		0.61
3′-***AUUACGAUUAUACAUCCUCA	centrocyte - tonsils	0.29
3′-***CUUACGAUUAUACAUCCUC	plasma cell - tonsils	0.28
3′-ACAAUUACGAUUAUACAU****	prostate	0.27
3′-****UUACGAUUAUACAUCCUC	peripheral blood mononuclear cells	0.05

**Figure 3.**
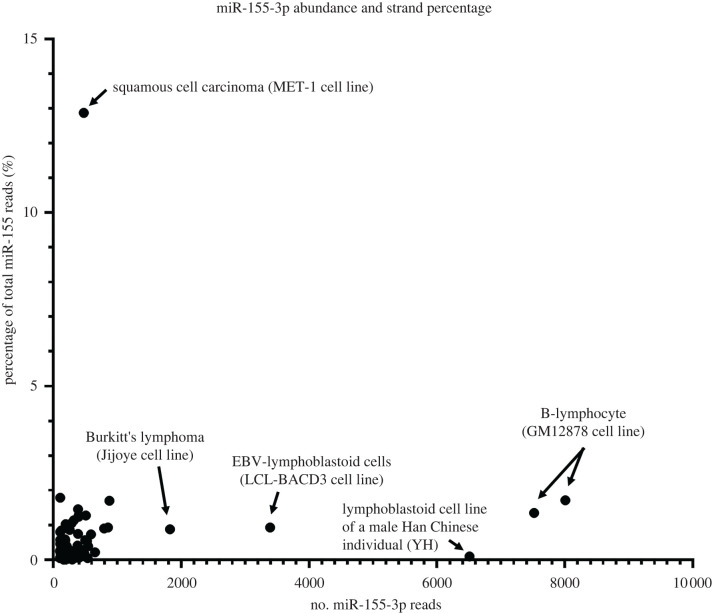
Comparison of miR-155-3p abundance and strand percentage. miR-155-3p abundance and strand percentage of total miR-155, derived from miRSwitch data [95]. Labelled are examples of cells showing either high miR-155-3p abundance but low strand percentage of total miR-155 strand reads or high percentage of the miR-155-3p strand.

A systematic analysis of arm switch events from high-throughput expression data can be conducted using miRSwitch, a tool that utilizes publicly available miRNA sequencing data to identify the abundance of miRNA-5p and -3p strands in various tissues and conditions [95]. Within these publicly available datasets, there are few examples of true arm switching events occurring (figure 3). B-lymphocytes show the highest expression of miR-155-3p (8009 reads). However, this represents only 1.71% of the total miR-155 strand reads. The majority of datasets showing significant miR-155-3p expression are similar, doing so as a less than 2% fraction of the total miR-155 strand population (figure 3). From this, it is tempting to conclude that miR-155-3p synthesis is unlikely to be a regulated event, but rather a by-product of excessive miR-155-5p synthesis due to natural imprecisions in processing. However, such conclusion is somewhat contradicted by the cutaneous squamous cell carcinoma dataset, wherein 478 reads of miR-155-3p are found, accounting for approximately 12.9% of the total miR-155 strand population. Thus, the concentration of miR-155-3p isomiRs and the increased miR-155-3p strand percentage in cancerous tissues may indicate a specific dysregulation of miR-155 processing within these conditions.

### 6.5. miR-155-3p conservation

**Figure 4.**
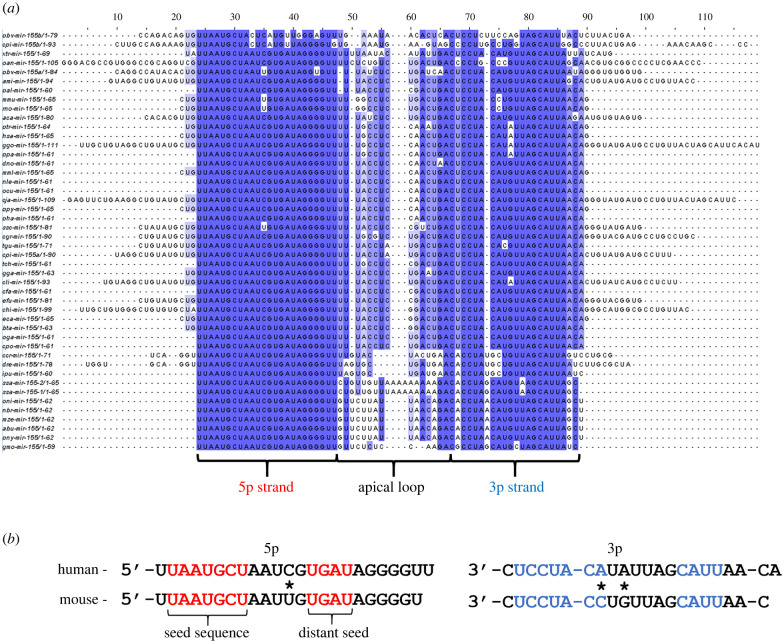
miR-155 evolutionary conservation and human/murine miR-155 strand comparison. (*a*) Pairwise alignment of pre-miR-155 strands collected from miRBase v22 and visualized using Clustal Omega software. (*b*) Direct comparison of human and murine miR-155 strands, with the seed sequences (red for the 5p strand and blue for the 3p strand) and strand differences (*) labelled.

Multi-species sequence alignment shows that miR-155 has a general high level of conservation across mammalian species (figure 4*a*). As expected, the miR-155-5p strand remains largely unchanged, an observation indicative of its conserved functionality. However, following general observations of miRNA* strand evolution, the miR-155-3p sequence shows a greater degree of variation (figure 4*a*) [96]. Firstly, an A at position 10 of the 3p strand occurs in apes, this seeming to correct the U·G wobble base pair found in other mammals at this position (figure 4*b*). Of predicted greater consequence is the occurrence of a C at position 8 of the miR-155-3p strand in mice and rats (figure 4*b*). This change affects the final nucleotide of the core seed sequence, thus likely leading to some divergence between murine and human mRNA target repertoire and resultant miRNA strand functionality [10]. In addition, variation outside of the nucleotides constituting the mature miR-155 strands has the potential to affect pri-miR-155 processing, as in the case of miR-10 whereby a change outside of the mature sequence is the most likely cause of its differing strand preference in *Drosophila* and *Tribolium* [58]. Notable changes include the enlarged murine miR-155 apical loop, 13 nt compared to the 4 nt human apical loop, the predicted 1 nt overhangs produced by Dicer and Drosha cleavage, and the upstream migration of the mGHG and basal UG motifs, all contributing to potential changes to murine pri-miR-155 processing (figure 2) [80,82–84]. Notably, miRBase lists the murine miR-155-3p sequence with the highest confidence as having approximately 10-fold higher expression (10.9 reads per million) than that of human (1.97 reads per million) [97]. These potential differences between mouse and human miR-155 processing and functionality must be taken into consideration when analysing and interpreting the evidence for miR-155-3p functional relevance.

## 7. MiR-155-3p functionality

To date, miR-155-3p is not well represented in the literature, likely due to a combination of assumed miRNA* strand non-functionality, low expression levels thwarting detection, and it being overshadowed by its highly expressed and functionally well characterized partner miR-155-5p strand. The latter regulates the immune system and is implicated in a range of pathologies such as rheumatoid arthritis, multiple sclerosis, infection and cancer [85,86,98]. Irrespective of any assumed miRNA* strand non-functionality, miR-155-3p has been functionally investigated and implicated in a handful of biological processes, including the immune response, cardiac remodelling and cancer, and these studies are summarized in tables 2 and 3.

**Table 2.** miR-155-3p mRNA targets and experimental validation. Overview of reported miR-155-3p targets with type of experimental validation. GFP, green fluorescence protein; NP, nucleus pulposus.

target (ref)	species	cell line	disease/process	function	miR-155-3p target validation
**KCTD1** [99]	mouse	cementoblasts (OCCM-30)	periodontitis	enhancer of β-catenin degradation	luciferase assay, miR-155-3p mimic/overexpression and inhibition
**TP53INP1** [94]	human	lung cancer cells (A549 and NCI-H1975)	adenocarcinoma	tumour suppressor; induces G1 arrest and p53-mediated apoptosis	luciferase assay, miR-155-3p mimic/overexpression and inhibition
**SIX1** [100]	human	glioblastoma cells (U87 and A172)	glioma	oncogene; inhibits apoptosis and modulates cell cycle regulators	luciferase assay, miR-155-3p mimic/overexpression and inhibition
**PCDH7** [101]	human	U87 and primary glioblastoma cells	glioma	suppressor of the Wnt/β-catenin pathway	luciferase assay, miR-155-3p mimic/overexpression and inhibition
**CREBRF** [102]	human	glioblastoma cells (U251 and T98G) and primary glioma cells	glioma	inhibitor of CREB3	luciferase assay, miR-155-3p mimic/overexpression and inhibition
**MYD88** [93]	human	MCF-7 cells	breast cancer	transducer of TLR signalling, promoting NF-kB and AP-1 activity	luciferase assay, miR-155-3p mimic/overexpression and inhibition
**CADM1** [91]	human	MCF-7 cells	breast cancer	anti-metastasis adhesion molecule	luciferase assay, miR-155-3p mimic/overexpression and inhibition
**MEF2C** [103]	mouse	embryonic stem cells	cardiac remodelling	pro-cardiogenesis transcription factor	luciferase assay, miR-155-3p mimic/overexpression and inhibition.
**FBXW7** [104]	human	BEL-7405 cells	hepatocellular carcinoma	component of the ubiquitin proteasome system	luciferase assay, miR-155-3p mimic/overexpression and inhibition
**Dnaja1/Dnajb2** [105]	mouse	EAE CD4^+^ T cells	experimental autoimmune encephalomyelitis (EAE)	heat-shock protein shuttling and localization	luciferase assay, miR-155-3p inhibition
**LT-β** [106]	human	B cell lymphoma (REC-1)	mantle cell lymphoma	activates the non-canonical NF-kB pathway	luciferase assay, miR-155-3p mimic/overexpression
**IRAKM** [107]	human	primary dendritic cells	inflammatory response	inhibitor of TAK1 dependent NF-kB activation	luciferase assay, miR-155-3p mimic/overexpression and inhibition
**PTEN** [108]	human	trophoblast cells (HTR-8/SVneo)	inflammatory response	tumour suppressor; activates PI3 K/Akt signalling	GFP assay, miR-155-3p mimic/overexpression and inhibition
**NKIRAS1** [108]	human	trophoblast cells (HTR-8/SVneo)	inflammatory response	inhibits IkB degradation	GFP assay, miR-155-3p mimic/overexpression and inhibition
**GAB2** [109]	mouse	Raw264.7	inflammatory response	activates PI3K/Akt, JAK-STAT and JNK/SAPK signalling	luciferase assay, miR-155-3p mimic/overexpression
**WDR82** [110]	human	SW620 cells	colorectal cancer	aids in targeting of H3-Lys4 trimethylation	luciferase assay, miR-155-3p mimic/overexpression
**KDM3A** [111]	human	primary NP cells	intervertebral disc degeneration	regulator of histone modifications	luciferase assay, miR-155-3p mimic/overexpression

**Table 3.** miR-155-3p disease associations. Overview of studies associating upregulation or downregulation of miR-155-3p expression to a disease. n.a., data not available.

condition (ref)	species	sample	method	miR-155-3p change	miR-155-5p change
**inflammatory disorders**					
experimental autoimmune encephalomyelitis (EAE) [105]	mouse	miR155HG knockout spleen and brain samples of EAE mice	qRT-PCR and flow cytometry	↑	↑
multiple sclerosis [112].	mouse	cuprisone treated mice	qRT-PCR	↑	n.a.
lung infection [113]	mouse	lung infected with wild bird influenza A virus subtype H5N2	microarray and qRT-PCR	↑	—
lung injury [114]	mouse	lungs treated with LPS	microarray and qRT-PCR	↑	↑
**cancer**					
non-small cell lung cancer (NSCLC) [115]	human	62 NSCLC tumour tissues	qRT-PCR	↑	↑
glioma [100,102]	human	40 glioma samples and 5 secondary cell lines	qRT-PCR	↑	n.a.
	human	hypoxic U251 and T98G cells	microarray, qRT-PCR	↑	n.a.
renal cell carcinoma (RCC) [90]	human	4 secondary RCC cell lines	qRT-PCR	↑	↑
hepatocellular carcinoma [104]	human	45 paired tissues and secondary cell lines	qRT-PCR	↑	n.a.
colorectal cancer [110]	human	46 paired tissues	qRT-PCR	↑	n.a.
mantle cell lymphoma [106]	human	mino secondary cell line	microarray	↑	—
breast cancer [91–93]	human	1103 primary tumours / 7 paired tissues	microarray/qRT-PCR	↓	n.a.
	human	128 paired tissues	qRT-PCR	↑	n.a.
	human	131 paired tissues	microarray/qRT-PCR	↑	n.a.
**other disorders**					
pulmonary silicosis [116]	rat	lung fibroblasts from 24-week silica treated animals	qRT-PCR	↑	n.a.
asthma [117]	mouse	whole lung treated with ovalbumin	qRT-PCR	↑	↑
bipolar disorder [118]	human	lithium responsive lymphoblastoid cell lines	microarray/qRT-PCR	↑	—
intervertebral disc degeneration (IDD) [111]	human	36 IDD nucleus pulposus cell tissues	qRT-PCR	↓	n.a.

## 8. The immune function of miR-155-3p

### 8.1. Dendritic cells

**Figure 5.**
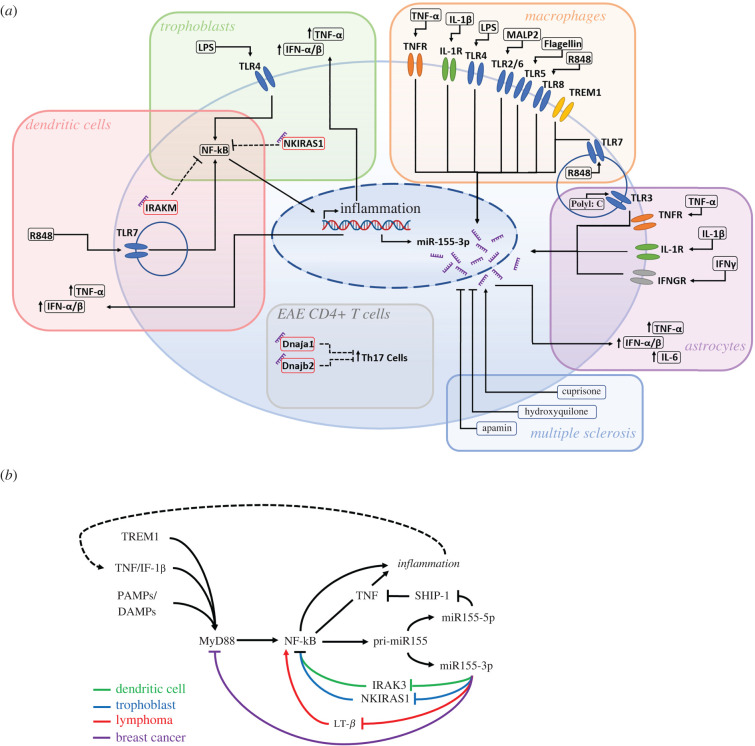
Inducers and targets of miR-155-3p in immunity. (*a*) Inducers, targets and phenotypic effects of miR-155-3p in specific cellular or pathological contexts, including dendritic cells [107], trophoblasts [108], macrophages [114,119], astrocytes [120], CD4+ T cells [105], EAE and multiple sclerosis [112,121]. (*b*) Graphic representation of positive and negative inflammatory feedback loops of miR-155-3p within the NF-κB pathway. Colours are used to indicate the cellular context of the validated pathways in dendritic cells [107] (green), trophoblasts [108] (blue), lymphoma [106] (red) and breast cancer [93] (purple).

The first biological function of miR-155-3p was identified by Zhou *et al*. [107], wherein stimulation of TLR7 in human plasmacytoid dendritic cells (pDCs) resulted in the induction of both miR-155 strands at levels above other miRNAs, an upregulation which was attributed to the c-Jun N-terminal kinase pathway (figure 5*a*) [107]. Interestingly, each miR-155 strand was linked to opposing functions within the pDC, with each being upregulated at different stages of activation. Specifically, miR-155-3p expression peaked at 4 h after stimulation and was found to target the TLR pathway inhibitor interleukin-1 receptor-associated kinase M (IRAKM), thus promoting expression of cytokines such as TNF and IFN-α/*β* (figure 5*b*). Conversely, miR-155-5p reached its peak induction 12 h after stimulation and was found to target the TLR signalling component TAB2, thus reducing cytokine expression. Asynchronously, but cooperatively, miR-155-3p and miR-155-5p coordinated the transient induction of IFN-α during pDC activation, with induction of miR-155-3p causing IFN-α upregulation in the early stages of the response, before being eclipsed by the induction of miR-155-5p, which later attenuated IFN-α expression. Inhibition of IRAKM by miR-155-3p has also been verified in lipopolysaccharide (LPS) induced third trimester trophoblasts, alongside its targeting of NF-kB inhibitor-interacting Ras-like 1 (NKIRAS1) (figure 5*b*) [108]. This was observed to create a positive feedback loop, whereby inhibition of IRAKM and NKIRAS1 expression reduced their suppression of the transcription factors NF-kB and AP-1. In turn, the increased activity of these transcription factors downstream of the TLR4 pathway led to increased expression of miR-155-3p, whose host gene contains promoters for the transcription factors [107,108,122]. Such an increase via LPS stimulation is mirrored in a separate trophoblast study of miR-155-5p [122]. Interestingly, across these studies an asynchronous induction of the two miR-155 strands is witnessed similarly to that observed in pDCs, with miR-155-3p and miR-155-5p induction peaking during the early LPS response (less than 6 h) and the late response (greater than 12 h), respectively.

Build-up of the Drosha and Dicer associated factor KH-Type Splicing Regulatory Protein (KHSRP) was shown to partially contribute to the staggered expression of the miR-155 strands in pDCs, with its knockdown decreasing miR-155-5p expression while increasing that of miR-155-3p and pri-miR-155 [107]. It is thought that KHSRP may promote miR-155 processing efficiency, thus leading to increased quantities of the primary miR-155 biosynthesis product miR-155-5p, and reducing the quantity of unprocessed pri-miR-155. This process could be aided by the miR-155-3p/NF-kB/AP-1 positive feedback loop, which, although experimentally validated only in trophoblasts, may occur also in pDCs due to the TLR7 pathway also culminating in the recruitment of these transcription factors to specific target genes [123]. The positive feedback loop may facilitate the initial fold increase in miR-155-3p by enhancing pri-miR-155 transcription in an environment lacking co-factors necessary for efficient processing. However, as type 1 IFNs have been shown to increase KHSRP expression, after this initial spike the KHSRP activity is sufficient to cause the strand induction ratios to switch, with miR-155-5p levels rising and miR-155-3p levels falling [107]. Of note is that KHSRP has also been shown to be a key factor in the processing of miR-155-5p in macrophages, with its depletion impairing the induction of miR-155-5p following LPS treatment and showing a concomitant increase in pri-miR-155 [124]. Although this study has not measured miR-155-3p levels, KHSRP, pri-miR-155 and miR-155-5p expression patterns are similar to those seen in pDCs, suggesting that KHSRP may be involved in miR-155-3p arm switching in the wider immune context.

### 8.2. Macrophages

The functionality of miR-155-5p in macrophages in various inflammatory contexts has been extensively studied. Mature miR-155-5p is one of the first effectors of TLR4 signalling, being strongly induced within 2–4 h after LPS treatment and having an overall net pro-inflammatory effect. It promotes the production of key pro-inflammatory cytokines such as TNF-α either directly, by an unknown mechanism, or indirectly, for example by inhibiting the anti-inflammatory regulator SH-2 containing inositol 5′polyphosphatase 1 (SHIP1) [8,125–128]. miR-155-5p has also been shown to promote a pro-inflammatory phenotype via IL-13R*α* inhibition, leading to M1 macrophage polarization, and inhibition of suppressor of cytokine signalling 1 (SOCS-1), promoting the action of the STAT pathway [129,130].

By contrast, miR-155-3p has been underinvestigated in macrophages. The first of few studies was conducted by Yuan *et al*. [114], whereby miR-155-3p/5p were both shown to have impaired expression in a microarray analysis of macrophages featuring a triggering receptor expressed on myeloid cells 1 (TREM-1) knockdown [114]. This cell surface receptor is an amplifier of TLR induced inflammation that triggers a signalling cascade which promotes NF-kB activation and resultant cytokine expression [131–133]. It was found that the pro-inflammatory activity of TREM-1 was in part due to its induction of miR-155 expression, probably due to the positive feedback of miR-155-5p into the pro-inflammatory TLR signalling cascade and possibly also due to a cooperative activity of miR-155-3p. Notably, in an experiment examining the effect of an NF-kB inhibitor on miR-155 levels during LPS induction or TREM-1 activation, miR-155-5p expression displayed a dramatic fold decrease with either treatment and NF-kB inhibition, returning to levels similar to basal [114]. However, miR-155-3p exhibited expression profiles that were clearly different to those of its partner strand. Stimulation of macrophages with LPS yielded a miR-155-3p induction of approximately 25-fold, compared to TREM-1 activation which yielded an induction of only approximately 2.5 fold. This is a remarkable difference, given that TREM-1 and the TLR4 pathways share common transcriptional effectors and the fold changes in miR-155-5p expression were approximately 2.5 and approximately 1.8, respectively. In addition, inhibition of NF-kB caused only a marginal decrease in LPS-induced miR-155-3p expression (down from approx. 25 to 20-fold change) compared to miR-155-5p expression whose induction was completely prevented. It is not possible to draw firm conclusions from these results as relative and not absolute miRNA abundance is reported. However, this may suggest that induction of miR-155-3p expression during the inflammatory response of macrophages is not simply a by-product of normal miR-155-5p processing as, if that were the case, the increase in both strands, with either TREM-1 or LPS as activator, would be proportional. Furthermore, the high disproportionality in strand induction following inhibition of NF-kB, with miR-155-3p induction remaining high and miR-155-5p being suppressed, lends itself to the speculation that miR-155-3p induction via LPS is a result of a post-transcriptional regulatory mechanism outside of the classically induced TLR4 pathway.

Later, Simmonds [119] further investigated the role of miR-155-3p in macrophages, overcoming previous flawed approaches of miRNA fold induction measurements by performing absolute quantification of the miR-155 strands alongside examination of the functionality of miR-155-3p [119]. Here, a staggered induction of the miR-155 strands like that occurring in pDCs and trophoblasts was reported, with miR-155-3p induction being detectable 20 min after LPS stimulation and reaching its peak 2–4 h later, and miR-155-5p peaking at approximately 8 h. Similar fold-increases in miR-155-3p were detected using a variety of inflammatory ligands, including TNF-α (TNFR), IL-1*β* (IL-1R), MALP2 (TLR2/6), Flagellin (TLR5) and R848 (TLR7/8), in addition to LPS. Interestingly, poly(I:C) (TLR3) and Pam3Cys (TLR1/2) did not produce an equivalent induction (figure 5*a*). Overall, this further attests to the notion that miR-155-5p and miR-155-3p expression poorly correlate and may be controlled by distinct immune regulatory pathways [124]. Of note in this study is also the induction of miR-155-3p that seems to co-occur with that of pri-miR-155, contrasting miR-155-5p expression, which increases steadily and continues to rise while pri-miR-155 levels decline. However, as the qRT-PCR analysis of pri-miR155 expression following LPS stimulation was performed using GAPDH as endogenous control, a housekeeping gene with well documented variability in most experimental conditions including the inflammatory response, the relationship between miR-155-3p and pri-miR-155 expression requires further investigation [134–136].

The miR-155 strands respective copy number per cell lends a degree of context to the miR-155-3p/5p biological functionality and the presence of a possible arm switching event. At rest, the quantity of miR-155-5p was found to dwarf that of miR-155-3p, with a copy number per cell of 1315 (±417) and 29 (±11), respectively. The accepted threshold for miRNA functionality is approximately 1000 copies per cell, as stated by TargetScan, meaning that in resting macrophages miR-155-3p has potentially minimal biological effect. At its highest fold increase at 2 h post LPS induction, miR-155-3p reaches a copy number of 767 (±137), a functional quantity, however still overshadowed in abundance by the 5578 (±1361) copy number per cell of miR-155-5p, which continues to increase as miR-155-3p copy number quickly decreases. This portrays a very different relationship between miR-155-5p and miR-155-3p than that shown within this and previous studies reporting fold of induction, with it not being a separate temporal induction of the two strands but instead the induction and attenuation of the miR-155-3p strand, while miR-155-5p continues to be expressed at increasingly high and functionally relevant quantities throughout. It has been noted during investigations into miRNA half-lives that miR-155-5p has a considerably longer half-life of approximately 10.5 h compared to that of miR-155-3p of approximately 4 h. This is predicted to be due to the weaker binding of the passenger strands to AGO, but also could be attributed to the increased association of the greater expressed 5p strand with mRNA providing strand stability via TMMP [137,138]. Although such studies of miRNA decay were not conducted in an immune context, they still provide insight into the relative stability of the two strands, with this possibly accounting for the fast attenuation of miR-155-3p in macrophages after its induction at approximately 2 h post LPS stimulation as witnessed by Simmonds [119]. Thus, the miR-155-3p strand is potentially active during the earliest stage of the inflammatory response, before settling back into levels of expression below a biologically relevant threshold.

Absolute quantification methods also illuminate issues with the aspersion that low abundance miRNAs, usually the miRNA* strand, are non-functional, as here the highly dynamic nature of miRNA regulation is evident, with miR-155-3p only existing at a presumed functional abundance in activated macrophages during a narrow time frame [119]. Following this experiment, Simmonds also investigated the functionality of miR-155-3p, showing that 15% of the approximately 767 copies per cell (approx. 115 copies in total) input associated with the RISC complex at 2 h post LPS induction. Interestingly, only approximately 4.5% of the miR-155-5p input of 5576 copies per cell (approx. 250 copies in total) were incorporated in the RISC complex at the same time point. Together, this further validates some degree of functionality of miR-155-3p at this early stage of the immune response.

Using miRNA binding site prediction, Simmonds [119] identified a target site for miR-155-3p in the 3′ UTR of the proinflammatory cytokine TNF-α. Notably, upregulation of TNF-α has been previously attributed to miR-155-5p, with inhibition of the miRNA leading to TNF-α attenuation during its usual rapid induction in the early stages of the macrophage response to LPS [139–141]. Various hypotheses exist as to how the miR-155-5p causes a direct upregulation of TNF-α translation, but none has been validated, especially as no binding sites for miR-155-5p on the TNF-α mRNA have been found. Thus, miR-155-3p presents a potential means whereby miR-155 may directly upregulate TNF-α translation by influencing its mRNA structure, either blocking its self-inhibition or increasing its stability [139,140]. However, Simmonds [119] discredited such a notion, finding no significant change in TNF-α protein production or mRNA levels following inhibition of either miR-155 strand, a result that is at odds with other *in vitro* and *in vivo* studies providing evidence for miR-155-5p- and -3p-mediated regulation of TNF-α [109,139–141].

While the work by Simmonds [119] undertook the deepest examination of macrophage miR-155-3p activity to date, it also presents a number of limitations which require further investigation to validate the findings reported. Such caveats include a microarray analysis of LPS stimulated monocyte-derived macrophages that shows only five miRNAs with a greater than 1.5-fold upregulation and no downregulated miRNAs, which is at odds with previous studies, as well as the aforementioned use of GAPDH as an internal control gene for qPCR, and the utilization of an immune stimulatory lipofection reagent [134,142,143].

### 8.3. Astrocytes

In 2012, Tarahassishin *et al*. [120] published a similar finding to the preceding study in pDCs, finding that miR-155-3p was the miRNA with the greatest induction in activated human foetal astrocytes treated with IL-1-β or IFN-γ (figure 5*a*). This study also provided the first evidence of cooperative roles for both strands of miR-155 in the induction of the inflammatory response, as opposed to the non-cooperative functionality witnessed in pDCs [107]. Separate 24 h treatments with pro-inflammatory cytokines and TLR ligands such as TNF-α, poly(l:C) and IL-1-β, both alone and with IFN-γ, all gave rise to large miR-155-3p/5p fold inductions (figure 5*a*). Meanwhile, treatment with miR-155-3p and miR-155-5p inhibitors implicated both strands in the upregulation of TNF-α, IL-6 and IL-8 in IL-1-β/IFN-γ activated astrocytes (figure 5*a*) [120]. Together, these results implicate miR-155-3p in the process of induction of inflammation alongside its partner strand, with astrocyte activation increasing expression of both miRNA strands that in turn promotes pro-inflammatory cytokine expression and the perpetuation of the inflammatory response. This is a process which had previously been identified in macrophages, with only miR-155-5p examined, and which aligns with a subsequent study identifying miR-155-3p/5p as both being significantly expressed in the inflammatory M1 polarized macrophage [119,144].

Recently, miR-155-3p has been implicated in the central nervous system (CNS) chronic inflammatory disorder multiple sclerosis (MS), with treatment of mice with the pro-demyelination agent Cuprisone leading to elevated levels of the miRNA strand (figure 5*a*) [144]. Treatment with the anti-malarial drug hydroxychloroquine saw attenuation of the MS phenotype as well as halving miR-155-3p expression [112]. Meanwhile, an independent study showed Apamin treatment to reduce miR-155-3p expression below that of the healthy control during the demyelination phase (figure 5*a*) [121]. Both treatments are known to target and inhibit the activity of microglial cells, possibly signifying that miR-155-3p plays a wider role in the CNS inflammatory landscape beyond that previously documented in astrocytes [120]. However, the rationale for investigating miR-155-3p in these studies is unclear, with no comparison to miR-155-5p expression and no attempt to functionally characterize the role of the 3p strand in MS. Beyond possible interactions with microglia, miR-155-3p has also been identified to play a role in MS via its expression by T cells, in a study which more rigorously defines the miRNA strands mechanistic involvement in this chronic inflammatory disease [105].

### 8.4. T cells

One of the core functions of miR-155-5p in immunity and inflammation is the regulation of T cells. Specifically, miR-155-5p has been found to induce proliferation and differentiation of both Treg and Th17 cells, which act to suppress and promote the inflammatory response, respectively [145]. A potentially related function has been identified for miR-155-3p in one of its few published investigations in T cells, in which CNS-infiltrating T cells are examined during murine experimental autoimmune encephalomyelitis (EAE) [105]. In this model, it was found that miR-155-3p mimics promoted the upregulation of the Th17 marker genes RORA and IL17A in CD4^+^ T cells (figure 5*a*). This being accomplished through the direct inhibition of the heat shock proteins Dnaja1 and Dnajb2, which regulate these Th17 markers [105]. Correspondingly, miR-155-3p inhibition showed that miR-155-3p had a greater influence on these markers when compared to its partner strand, irrespective of the fact that its molecular copy number was far lower than that of miR-155-5p. This highlights that the miRNA-3p copy number may not necessarily be indicative of the magnitude of its functionality, as it is the activity of the mRNA targets, in this case, Dnajb1 and Dnaja2, which ultimately demonstrates the biological relevance of the miRNA. This role of miR-155-3p was not found in healthy mice, thus may present a pathway only active during CD4^+^ autoimmune demyelination of the CNS [105]. EAE has been linked to significant changes in RISC assembly, with downregulation of Ago2 and a reduction in co-factor interactions leading to a decrease in miRNA-RISC binding [146]. Such destabilization of the RISC complex has the potential to cause an arm shift in favour of miR-155-3p, introducing inaccuracy in miRNA processing while miR-155 transcription in the CNS-infiltrating T cells is heightened.

### 8.5. miR-155-3p in inflammation: summary

Together, the studies published so far, including those discussed here, provide evidence that miR-155-3p is functionally relevant in the immune context as a pro-inflammatory regulator in multiple immune cells, including dendritic cells, macrophages, T cells and astrocytes. Immune responsive miRNAs such as miR-155-5p play an important role in the dynamic regulation of inflammatory signalling, as they can target multiple transcripts at the same time and their biogenesis does not require protein synthesis, thus allowing cost-effective and rapid amplification or suppression of cellular signals that fine-tune immune responses. These attributes are of extreme importance in inflammatory signalling, where misregulation of secreted factors can lead to widespread tissue damage in autoimmune disease and chronic inflammation. Interestingly, time course analyses of miRNA strands expression show that the induction of miR-155-3p appears to be limited to the early immune response, possibly indicating a conserved functionality for the miRNA within this timeframe, before the induction of its partner strand occurs. Such a function could lie in the positive feedback loops established between miR-155-3p and the NF-kB signalling pathway (figure 5*b*). Specifically, by suppressing NF-kB inhibitors, miR-155-3p may be acting to remove a regulatory checkpoint that could prevent a fast and strong inflammatory response, facilitating the rise in miR-155-5p, which mainly acts to enhance pro-inflammatory downstream signals such as TNF-α.

## 9. miR-155-3p in cancer

miR-155-5p is one of the most studied oncogenic miRNAs, with its dysregulated expression consistently identified in both solid and haematological cancers, including lung, breast, pancreatic, gastric, colorectal and endometrial cancers as well as melanoma, glioblastoma and osteosarcoma [147–155]. miR-155-5p plays two broad functions in cancer. First, it regulates the tumour microenvironment and the immune response to cancer, having pro- or anti-tumour effects depending on the immune cell type in which it is upregulated. Second, miR-155-5p expression associates with cancer drug resistance, with administration of miR-155-5p being shown to reduce drug effectiveness [156,157]. The breadth and depth of the investigation of miR-155-5p in cancer research has also shed light on miR-155-3p, which has been implicated in multiple types of cancer.

### 9.1. Lymphoma

**Figure 6.**
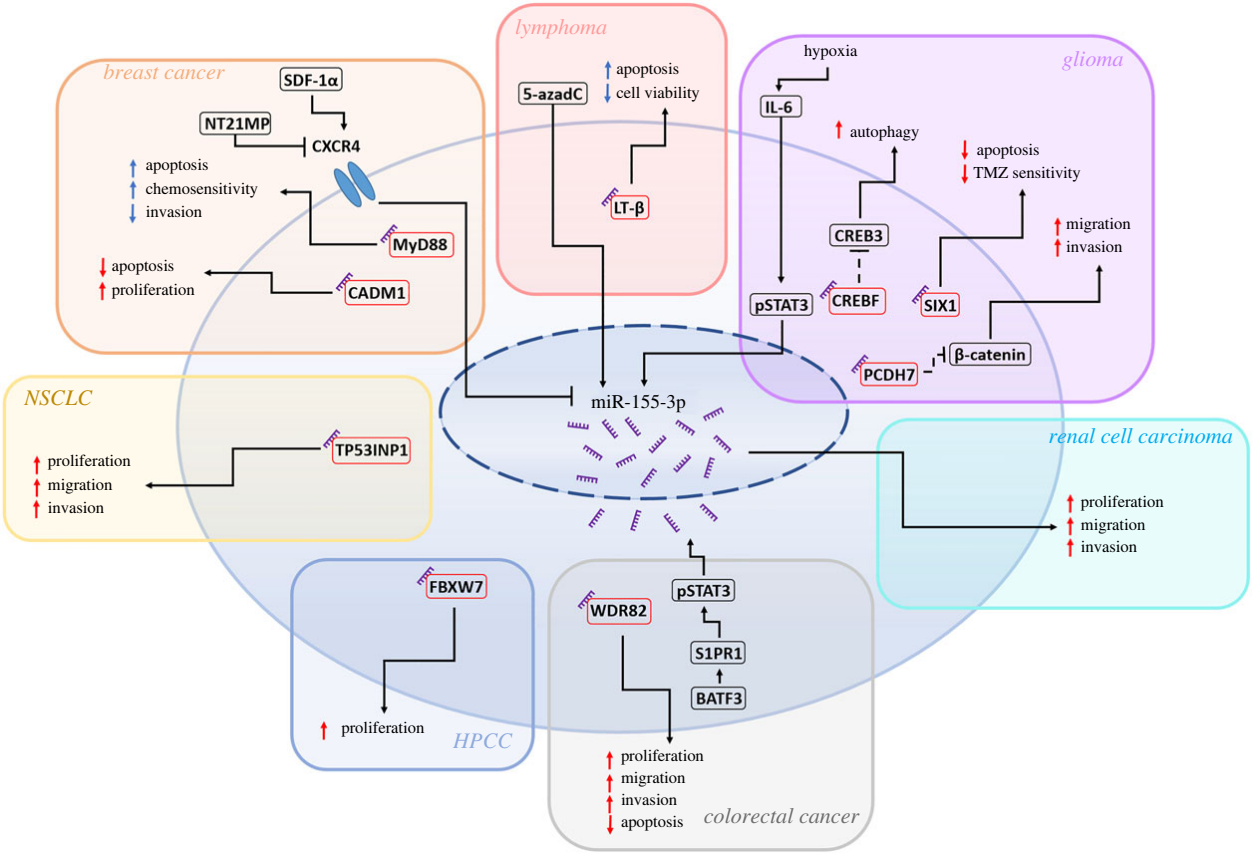
Targets and inducers of miR-155-3p expression in cancerous tissue. Inducers, targets and phenotypic effects of miR-155-3p separated by cancer cell type, including breast cancer [91,93,94,147], lymphoma [106], non-small cell lung cancer (NSCLC) [115], hepatocellular carcinoma (HPCC) [104], glioma [100–102], colorectal cancer [110] and renal cell carcinoma [90].

The first functional analysis of miR-155-3p in cancer was reported in 2014, wherein Yim *et al*. examined the role of the star-strand miRNA in mantle cell lymphoma [106]. Microarray analysis found a dramatic approximately 33-fold upregulation of miR-155-3p in the lymphoblast secondary cell line Mino that, remarkably, was accompanied by a lack of miR-155-5p upregulation. However, it must be noted that for the microarray analysis a fold change cut-off of 2.5 was set that could mask a more subtle, but still functionally relevant, miR-155-5p fold change, which is apparent in other lymphoblast contexts [158]. This discovery was followed by methylation analysis of the miR-155 host gene promoter, finding the promoter in Mino to feature a reduced degree of methylation compared to other lymphoma cell lines, with de-methylation via treatment with the DNA-hypomethylating agent 5-Aza-2′-deoxycytidine increasing miR-155-3p expression [106]. Further analysis of non-Hodgkin's lymphoma cell lines found miR-155-3p expression to inversely correlate with promoter methylation status, an interrelationship not found in miR-155-5p. Overexpression of miR-155-3p led to a reduction in lymphoma cell viability and an increase in cell apoptosis which was experimentally linked to the direct inhibition of lymphotoxin-β (LT-β) by miR-155-3p [106] (figures 5*b* and 6). LT-β had previously been demonstrated to be an oncogene thought to contribute to lymphomagenesis by activating the non-canonical NF-kB pathway and maintaining T and B cell localization [159,160]. Overall, this led to the authors defining miR-155-3p as tumour suppressive, an interesting hypothesis, especially when miR-155-3p is compared to its oncogenic partner miR-155-5p strand, that could infer an opposite action of the two strands in the context of lymphoma [158]. Theoretically, the miR-155 duplex could be creating a negative feedback loop, whereby the oncogenic overexpression of miR-155-5p, due to excessive NF-kB pathway activation, leads to increased expression of miR-155-3p, which in turn inhibits the induction of the non-canonical NF-kB pathway by LT-β. Such a relationship could potentially extend outside of the context of lymphoma and present an anti-inflammatory mechanism of miR-155-3p in the immune system as a whole.

### 9.2. Adenocarcinoma

miRNA expression analysis in non-small cell lung cancer (NSCLC) tissue found a greater relative induction of pri-miR155, miR-155-5p and miR-155-3p compared to neighbouring, healthy tissue [115]. Knockdown of pri-miR-155 expression led to a decrease in invasiveness, migration and proliferation, a phenotype that was rescued by transfecting either miR-155-5p or miR-155-3p mimics. The long non-coding RNA (lncRNA) TP53INP1 was identified as the likely causative target of both miR-155-5p and miR-155-3p, with overexpression and inhibition of the miRNA strands both showing an equivalent decrease or increase in TP53INP1 abundance [115]. TP53INPI was identified as a tumour suppressor in NSCLC, with its expression decreased in NSCLC tissue and negatively correlating with tumour grade. TP53INP1 has been implicated as a tumour suppressor in additional malignancies such as hepatocellular carcinoma (HPCC), breast cancer and pancreatic cancer, possibly providing scope for studying whether and how miR-155-5p/3p promote cancer in these contexts [161–163]. This mechanism strikingly depicts both strands of miR-155 acting in concert upon the same target with both seemingly having the same degree of effect, an unusual occurrence given the expected lower expression of the miRNA-3p strand.

### 9.3. Hepatocellular carcinoma

Expression of miR-155-3p has been found to be significantly increased within hepatic tumours, with a positive association with the miRNA strand found in late grade tumours as well as a decreased survival rate [104]. *In vivo* overexpression of miR-155-3p showed enhanced tumorigenesis, with an increase in tumour weight and colony formation, while inhibition of miR-155-3p elicited a reduction in these disease features to below that of the wild type. This effect of miR-155-3p was attributed to its direct inhibition of F-Box and WD Repeat Domain Containing 7 (FBXW7), an anti-proliferative protein whose overexpression and inhibition reversed or strengthened, respectively, the miR-155-3p overexpression phenotype. However, it should be noted that the overexpression method utilized in this study involved the transfection of a miR155-3p precursor, which could have also led to upregulation of miR-155-5p, potentially allowing either strand to be responsible for the effect of the overexpression.

### 9.4. Glioma

The expression of miR-155-3p and 5p shows significant association with increased glioma grade, with miR-155-3p also being positively associated with a reduction in survival rate, a correlation not shared by its partner strand [100,101]. Wu *et al*. [101] found that inhibition of miR-155-3p decreases invasiveness and migration of primary glioma cells, with overexpression of miR-155-3p, alongside inhibition of pri-miR-155, having the opposite effect [101]. However, miR-155-5p inhibition and overexpression was shown to have the same effect on invasiveness and migration, possibly illustrating a cooperative or compensatory functionality of the two strands in the primary cells line. More recently, inhibition of miR-155-3p in primary and secondary glioma cell lines was found to enhance cell apoptosis rate and reduce cell cycle progression, with the inhibited cells showing an increased sensitivity to the chemotherapeutic drug temozolomide (TMZ) [100]. *In vivo* work further verified this link, with miR-155-3p knockdown eliciting a decrease in tumour volume and increased TMZ sensitization.

Protocadherin-7 (PCDH7) and protocadherin-9 (PCDH9) were identified and experimentally validated as targets of miR-155-3p and miR-155-5p, respectively, within primary glioma cells [101]. Both protocadherins function as tumour suppressors in glioma, acting as inhibitors of the oncogenic Wnt/β-catenin signalling pathway via downregulation of β-catenin and cyclin-D [101]. Moreover, miR-155-3p has also been demonstrated to directly inhibit homeobox protein SIX1 in glioma, with reintroduction of SIX1 found to rescue the TMZ resistance induced by miR-155-3p mimic administration [100].

miR-155-3p expression is also upregulated in hypoxic glioma cells in a time and IL-6 dose dependent manner, with induction increasing up to 24 h and IL-6 inhibition causing attenuation of miR-155-3p expression [102]. Identification and experimental validation of a pSTAT3 binding site in the miR-155 host gene promoter shows a potential route for this upregulation, but also suggests that miR-155-5p may be upregulated too in these conditions due to the two miRNA strands sharing the primary transcript. Functionally, miR-155-3p was identified as a pro-autophagy regulator, with administration of a mimic eliciting a similar effect as that caused by exogenous IL-6, this being due to the miRNA directly inhibiting CREB3 regulatory factor (CREBRF), a negative regulator of the pro-autophagy transcription factor CREB3 [102].

### 9.5. Colorectal cancer

Upregulation of miR-155-3p via pSTAT3 activity is also apparent in colorectal cancer, with an approximately fourfold increase of miR-155-3p expression quantified within cancerous colorectal tissues and positively correlated with increased pSTAT3 levels [110]. Within colorectal cancer cells, miR-155-3p was found to directly inhibit WD repeat domain 82 (WDR82), a tumour suppressor which inhibits proliferation, migration, and invasiveness of colorectal tumours. In addition to providing a further mechanism of miR-155-3p acting as an oncogene, this study also highlights miR-155-3p induction via pSTAT3 as a cross-tissue occurrence. miR-155-5p expression has also been shown to be promoted by pSTAT3, creating a positive feedback loop whereby miR-155-5p directly targets suppressor of cytokine signalling 1 (SOCS1), a STAT3 inhibitor [147,164,165]. With the high degree of upregulation such a loop could achieve, it is therefore understandable that miR-155-3p could also exist at high levels and have a functional impact on the development of both colorectal cancer and glioma [102,110].

### 9.6. Breast cancer

In 2019, Zhang *et al*. conducted qPCR analysis of 128 paired breast cancer tissue samples, finding a significant increase in miR-155-3p expression, which associated with enhanced tumour progression and lower survival rate [91]. miR-155-3p inhibition and mimicry studies in MCF-7 cells and in *in vivo* xenograft models found that miR-155-3p acted to increase cell proliferation and repress apoptosis [91]. A further study identified the known tumour suppressor cell adhesion molecule 1 (CADM1) as a direct target of miR-155-3p repression, with overexpression of the protein abolishing the miR-155-3p tumorigenic phenotype.

However, this oncogenic model of miR-155-3p conflicts with an earlier study conducted by Lingyu Zhang *et al*. that identifies miR-155-3p as tumour suppressive [93]. Herein, a microarray analysis of 1103 breast tumour samples shows reduced expression of miR-155-3p compared to normal tissue. miR-155-3p was found to directly target and regulate the oncogene MyD88, this validating a previous study showing regulation of MyD88 by miR-155-3p, as well as also showing a limited degree of tropomyosin 1 alpha (TMP1) and interleukin-1 receptor-associated kinase 3 (IRAK3) modulation [93,94]. The central role of MyD88 in the TLR4 pathway further implicates miR-155-3p in inflammatory processes alongside its partner strand, although this anti-inflammatory function is contrary to a general trend of miR-155-3p exerting a pro-inflammatory function as outlined in this review.

miR-155-3p was further shown to decrease tissue invasion and cell migration, while increasing apoptosis in MCF-7 cells as well as decreasing tumour growth rate in *in vivo* xenograft models [93]. Interestingly, miR-155-3p overexpression was found to increase the cytotoxicity of the chemotherapy drug paclitaxel in resistant cells, a functionality opposed to the cancer drug resistance attributed to the miR-155-5p strand [93,156,157]. This and an earlier study both illustrate reduction of MyD88 as a result of miR-155-3p overexpression and the resultant effects, including decreased B-cell lymphoma 2 (Bcl-2) and increased bcl-2-like protein 4 (Bax) and caspase-3. These changes to oncogenic markers contrast those seen in the studies reporting an oncogenic function for miR-155-3p [93,94].

### 9.7. miR-155-3p in cancer: summary

Together, miR-155-3p is shown to be functional in a number of malignancies in different body locations and systems, with no discernible restrictions to tissue or cancer type. Primarily, miR-155-3p acts as an oncogene via the direct inhibition of tumour suppressors such as TP53INP1 (adenocarcinoma), FBXW7 (hepatocellular carcinoma), PCDH7/CREB3/SIX1 (glioma), WDR82 (colorectal cancer) and CADM1 (breast cancer) (figure 6) [92,93,100–102,104,110]. Within these malignancies, miR-155-3p is commonly associated with increased tumour grade and reduced survival rate, promoting a cellular phenotype of increased proliferation, migration and invasiveness while decreasing apoptosis. Notably, in two cases, miR-155-3p is described as tumour suppressive, as it directly inhibits the oncogenic LT-β (lymphoma) and MyD88 (breast cancer) [93,106]. Interestingly, regulation of these factors places miR-155-3p as an upstream regulator of NF-kB, forging a negative feedback loop, as NF-kB is a known promoter of pri-miR-155 synthesis. However, the miR-155-5p strand is known to create a positive feedback loop with NF-kB, via its inhibition of protein phosphatase 2 catalytic subunit alpha (PPP2CA), a negative regulator of Akt [166]. Thus, miR-155-3p has the potential to function as a buffer to the runaway expression of its partner strand, with the lower expression of miR-155-3p only being capable of halting miR-155-5p synthesis when the positive feedback loops have increased pri-miR-155 levels to a sufficient threshold as the initial inflammatory induction has ran its course.

## 10. Other roles of miR-155-3p

### 10.1. Bone growth and repair

**Figure 7.**
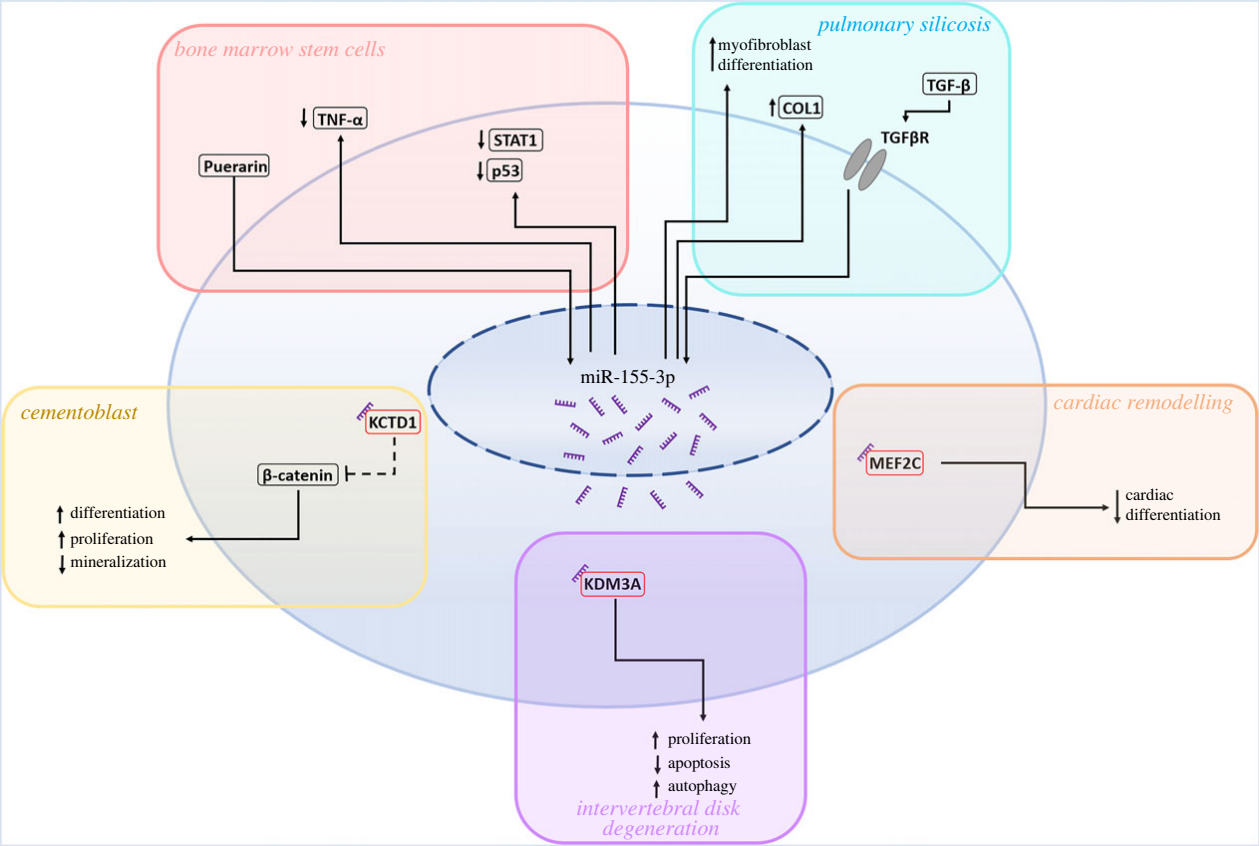
Targets and inducers of miR-155-3p expression in non-cancerous tissue. Inducers, targets and phenotypic effects of miR-155-3p are separated by cell type or disease, including bone marrow stem cells [168], cementoblasts [99], pulmonary silicosis [116], cardiac remodelling [103,169] and intervertebral disc degeneration [111].

MiR-155-3p, but not miR-155-5p, is upregulated in a rat model of spinal cord injury [167]. Knockdown of miR-155-3p in bone marrow stem cells led to an increase in p53, TNF-α and STAT1 levels, with these changes being rescued by the drug puerarin, which was shown to upregulate miR-155-3p levels [168] (figure 7). Puerarin administration promoted stem cell differentiation, bone formation and increased bone mass in murine bone grafts. This was reversed by miR-155-3p inhibition, indicating that miR-155-3p may play a role in the phenotype. These results match those seen within cementoblast cells, wherein miR-155-3p overexpression was found to promote a growth phenotype (figure 7) [99]. This being through suppression of mineralization via inhibition of alkaline phosphatase, osteopontin, osteocalcin, osterix and bone sialoprotein, and by upregulating β-catenin levels via direct inhibition of potassium channel tetramerization domain containing 1 (KCTD1), a protein which facilitates the degradation of β-catenin. This outcome is similar to the oncogenic effects of miR-155-3p in glioma where the miRNA enhances β-catenin signalling [101]. Furthermore, transfection of miR-155-3p mimic into intervertebral disc degeneration (IDD) model cells showed attenuation of the IDD phenotype, promoting proliferation and inhibiting apoptosis, while increasing autophagy rate with lysine demethylase 3A (KDM3A), identified as a potential causative target of the miRNA (figure 7) [111]. Overall, this solidifies the pro-growth functionality of miR-155-3p within a non-cancerous context, providing a basis from which miR-155-3p interactions in the context of cancer may have evolved.

### 10.2. Cardiac remodelling and lung fibrosis

During cardiac differentiation of embryonic stem cells, miR-155-3p is downregulated while miR-155-5p is notably upregulated [103]. Examination of miR-155-3p expression in foetal and adult rat cardiac remodelling shows decreased expression in both tissues, once again being the inverse of miR-155-5p, which is shown to be upregulated in both [169]. Inhibition of miR-155-3p increased the expression of cardiac specific markers while also increasing the expression of the cardiac morphogenesis factor myocyte enhancer factor 2C (MEF2C) in differentiated murine embryonic stem cells (figure 7) [103]. miR-155-3p was found to directly target MEF2C, leading to a decline in cardiac differentiation due to a reduction in MEF2C-mediated promotion of myogenesis gene expression. Cardiac remodelling presents an interesting case of miR-155-3p showing an inverse relationship to its partner strand both in functionality and in expression. Typically, increased miR-155-5p expression is accompanied by an increase in miR-155-3p, likely a by-product of enhanced biogenesis resulting from an increased rate of inaccurate cleavage or malfunctioning strand selection. However, in this instance, this inverse change in the strand ratio of the two miRNAs is instead indicative of a post-transcriptional event that directly orchestrates the expression of the two-strands.

Furthering our understanding of the role of miR-155-3p in growth and repair are findings in pulmonary silicosis wherein 24-week silica treatment of rats shows miR-155-3p as the only positively upregulated miRNA in isolated lung fibroblasts [116]. miR-155-3p was found to be induced in these cells by TGF-β1, with the upregulation in silicosis being implicated in an increase in collagen type I expression, indicative of increased extracellular matrix deposition, and the promotion of factors associated with myofibroblast differentiation (figure 7). This is not the only study of miR-155-3p in the lung, with miR-155-3p also being induced, alongside miR-155-5p, in a lung model of asthma in ovalbumin sensitized mice [117].

## 11. Future perspectives

### 11.1. Fold change versus functional abundance

The majority of the studies published so far have approached the investigation of the biological role of miR-155-3p by measuring its expression via qRT-PCR and utilizing fold change as a metric. This represents a technically straightforward and high-throughput method of assessing miRNA expression that is widely used in both mRNA and miRNA expression analyses. However, a flaw in this method becomes apparent when it is applied to measure the differential expression of miR-155-3p and other low-abundance miRNAs. Specifically, miRNA fold change is a metric that is not representative of miRNA molecular abundance as a large fold increase in a low-abundance miRNA, though appearing significant, may only represent a small increment in its abundance, the inverse being true for high-abundance miRNAs such as miR-155-5p. An example is provided in Mycko *et al.* (2015), whereby a qRT-PCR analysis of miR-155-3p and -5p levels in CNS-infiltrating CD4+ T cells during EAE is conducted [105]. This analysis shows an approximately 70-fold increase in miR-155-3p expression at day 13 with only an approximately 10-fold increase in miR-155-5p. When instead looking at copy number analysis of the same samples, the assumed relationship is notably reversed, with the induction of miR-155-3p only bringing it to a copy number of approximately 30 molecules per cell compared to approximately 120 molecules per cell of miR-155-5p. Moreover, further investigation in this study uncovers a functional role for miR-155-3p at this relatively low copy number. This further serves to highlight that the commonly low molecular abundance of miRNA* strands is not indicative of their functional activity, and that more thorough experimental approaches are necessary before these strands are discounted. For instance, the abundance of miRNAs within extracellular vesicles (EVs) should be measured as these low concentration miRNA ‘cargoes’ may be functional.

### 11.2. Experimental approaches

Absolute miRNA abundance measurement via the utilization of a qPCR standard curve has been used in several studies of miRNAs, and miR-155-3p specifically [105,119]. This grants an additional layer of insight into the functionality of low-abundance miRNAs and provides a better estimation of the functional impact of a miRNA on the cell compared to fold change measurement alone. Simmonds (2019) used immunoprecipitation of miRNA bound to the RISC complex followed by absolute miRNA quantification to determine the abundance of functional miR-155-5p/3p [119]. Such an approach exemplifies that total cell miRNA abundance is itself not indicative of the population functionally associated with the RISC complex.

Linked to this is the usage of miR-155-3p overexpression models without a tandem loss-of-function approach, an inappropriate common occurrence, which although granting insight into miR-155-3p potential targets, likely expresses the low-abundance miRNA at levels far higher than those that would naturally occur. In the case of miR-155-3p, an added problem arises as investigators often do not take into consideration the possible confounding effect of miR-155-5p upregulation on their study system. Upregulation of miR-155-3p usually occurs alongside that of its partner strand, which is normally more abundant. Therefore, extra care must be taken to accurately validate how miR-155-3p affects cell phenotype.

### 11.3. Arm switching and therapeutics

Manipulation of arm switching events provides an exciting avenue of potential disease treatment and diagnosis. For instance, it has been postulated that through the chemical inhibition of the arm switching associated factors TUT4/7, miR-324 strand ratio could be regulated to treat glioblastoma or HPCC [27]. Similarly, for miR-155, regulation of strand selection in favour of miR-155-3p could serve as a treatment for lymphoma, promoting the tumour suppressive 3p strand while the oncogenic 5p strand declines. However, before any therapeutic manipulation of miR-155 arm switching can be performed more research must be conducted into the post-transcriptional regulation of the miRNA as, due to the low expression of miR-155 outside of the inflammatory context, it is often ignored in large scale pri- and pre-miRNA processing screens.

### 11.4. Nomenclature

Discussion of miRNA* and miRNA-5p/3p illustrates the importance of nomenclature both for scientific accuracy and for accessibility, especially with the rise of digital databases and search engines which rely upon the consistent usage of key terms for information discoverability. With this being the case, it is concerning that publications focused on miRNAs still sometimes fail to clearly indicate which mature miRNA strands they are discussing, or measuring in experimentation. As previously outlined, the 5p/3p suffix ought to be used in all instances, with this giving the reader direct information on which miRNA sequence is being referenced as well as not including any biased assumption that abundance directly correlates to functionality. However, although the use of 5p/3p is becoming standard, many authors still do not indicate the miRNA strand at all. This is presumedly under the assumption that the reader will know they are discussing the higher expressed strand. This practice is as flawed as using the miRNA* label, and creates unnecessary and reduced reproducibility.

## 12. Conclusion

Considering the numerous examples of miR-155-3p functionality in both health and disease, any assumed non-functionality of low-abundance miRNA strands could be disputed in principle. Overall, miR-155-3p has been found to function within similar systems as its partner strand, promoting cell growth, cancer progression and the inflammatory response. Thus far, a mechanism for miR-155 arm switching has not been defined, with the current body of evidence indicating that miR-155-3p expression follows that of miR-155-5p, although at a generally lower abundance and with distinct expression kinetics. However, there are intriguing implications of miR-155-3p playing a regulatory role upon its partner strand, especially in an immune context, wherein miR-155-3p has been shown to inhibit NF-kB, an up-regulator of miR-155 expression that forms a positive feedback loop with miR-155-5p [93,165]. With the widespread implications of miR-155-5p in biological systems, future research shall begin to identify and characterize the likely multiple roles for its partner strand, with experimental approaches such as absolute miRNA measurement and in-depth temporal and spatial analysis of both strands, simultaneously enhancing the quality of studies produced.

## Data Availability

This article has no additional data.
